# SMARCD1 regulates senescence-associated lipid accumulation in hepatocytes

**DOI:** 10.1038/s41514-017-0011-1

**Published:** 2017-08-30

**Authors:** Chisato Inoue, Chong Zhao, Yumi Tsuduki, Miyako Udono, Lixiang Wang, Masatoshi Nomura, Yoshinori Katakura

**Affiliations:** 1 Graduate School of Bioresources and Bioenvironmental Sciences, Kyushu University, 6-10-1 Hakozaki, Higashi-ku, Fukuoka, 812-8581 Japan; 2 Graduate School of Systems Life Sciences, Kyushu University, 6-10-1 Hakozaki, Higashi-ku, Fukuoka, 812-8581 Japan; 3 Department of Medicine and Bioregulatory Science, Graduate School of Medical Science, Kyushu University, 3-1-1 Maedashi, Higashi-ku, Fukuoka, 812-8582 Japan; 4 Faculty of Agriculture, Kyushu University, 6-10-1 Hakozaki, Higashi-ku, Fukuoka, 812-8581 Japan

## Abstract

Previously, we have identified 16 senescence-associated genes by a subtractive proteomic analysis using presenescent and senescent human fibroblast cells, TIG-1. The aim of this study was to clarify the role of SMARCD1, one of the identified genes, also known as BAF60a, in hepatic senescence. SMARCD1 is a member of the SWI/SNF chromatin remodeling complex family, and regulates the transcription of target genes through the alterations of chromatin structure. We demonstrated that the reduced expression of SMARCD1 triggers cellular senescence and induces the accumulation of lipids, suggesting that SMARCD1 acts as a mediator in these processes. Furthermore, palmitic acid treatment and high-fat diet led to a significant reduction of SMARCD1 expression, and consequently induced cellular senescence and lipid accumulation in HepG2 cells and mouse liver, respectively. The results obtained here suggest that dietary nutrient-associated impaired expression of SMARCD1 triggers cellular senescence and lipid accumulation, indicating a potential application of SMARCD1 in the prevention of lifestyle-related diseases.

## Cellular senescence: SMARCD1, a key molecule in lipid accumulation

A team led by Yoshinori Katakura at Kyushu University tried to clarify the molecular mechanisms of pathogenesis of fatty liver, focused on SMARCD1, one of the identified senescence-associated genes, and revealed its pivotal roles in cellular senescence induction and lipid accumulation in hepatocytes. SMARCD1 is a member of the SWI/SNF chromatin remodeling complex family and known to regulate the transcription of target genes through the alteration of chromatin structure. The results obtained here suggest that dietary imbalance such as high-fat diet impaires expression of SMARCD1, which triggers cellular senescence and lipid accumulation in hepatocytes, indicating a potential role of SMARCD1 in the prevention of lifestyle-related diseases.

## Introduction

Various genes involved in many pathways have been associated with cellular senescence, including tumor suppressors (p53, p21, and p16), stress-related MAPK (p38), PI3K/AKT/mTOR, DNA damage response pathways, and molecules related to senescence-associated secretory phenotype (IL-6, IL-8, NF-κB, and c/EBPβ)^1–3^. Previously, using subtractive proteomic analysis, we have identified 16 senescence-associated genes in presenescent and senescent human fibroblast cell line, TIG-1, and SMARCD1 was selected for further investigations, representing the focus of this study^4^.

SMARCD1, also known as BAF60a, is a member of the SWI/SNF chromatin remodeling complex family, which regulates the transcription of target genes through binding to specific transcriptional factors and the alterations of local chromatin structure^5^. Several genes important for chromatin remodeling, such as p53 and Rb, affect cellular senescence by mediating changes in chromatin structure and gene expression^6^. Although SWI/SNF chromatin-remodeling family proteins regulate gene expression during cellular development as well and affect DNA damage response, their involvement in cellular senescence has not been reported previously^7^. Therefore, in this study, we evaluated the roles of SMARCD1 in cellular senescence and in senescence-related hepatocyte phenotypic changes.

## Results

### Functional role of SMARCD1 in cellular senescence

**Fig. 1:**
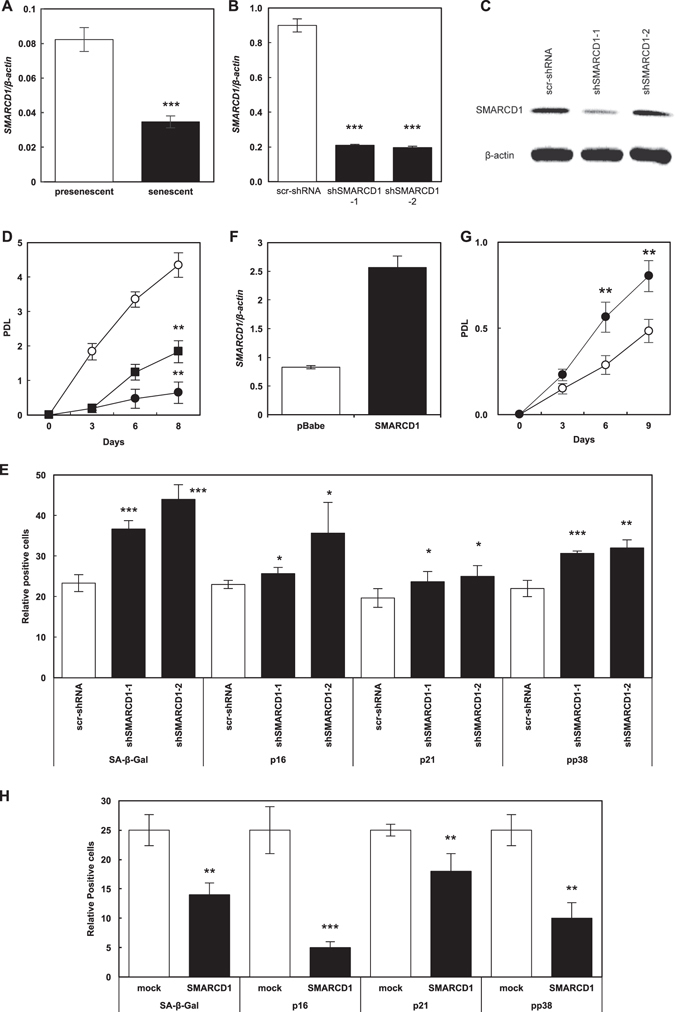
Functional analysis of SMARCD1 role in cellular senescence. Relative gene expression of *SMARCD1* in presenescnet and senescent TIG-1 cells (**a**), in scramble shRNA (scr-shRNA)-transduced and SMARCD1-silenced presenescent TIG-1 cells (**b**), and in mock-transduced and SMARCD1-overexpressing senescent TIG-1 cells (F). (**c**) Expression of SMARCD1 in scr-shRNA-transduced and SMARCD1-silenced presenescent TIG-1 cells. (**d**) Proliferation rate of scr-shRNA-transduced (○) and *SMARCD1*-silenced presenescent TIG-1 (●, shSMARCD1-1; ■, shSMARCD1-2). Cellular senescence phenotypes, quantitatively analyzed by IN Cell analyzer 1000. Activity and expression of senescence markers were measured in scr-shRNA-transduced and *SMARCD1*-silenced presenescent TIG-1 cells (**e**), and in mock-transduced and SMARCD1-overexpressing senescent TIG-1 cells (**h**). (**g**) Proliferation rate of mock- (○) and SMARCD1-transduced TIG-1 cells (●). Data were analyzed by two-sided Student’s *t*-test and expressed as mean ± SD. *P* < 0.05 was considered significant (^*^ *P* < 0.05; ^**^ *P* < 0.01; ^***^ *P* < 0.001)

The expression of *SMARCD1* was shown to be downregulated in senescent cells (Fig. 1a). Next, we evaluated the senescent phenotypes of presenescent TIG-1 cells (37 population doubling level (PDL)) after the expression of *SMARCD1* was reduced by shRNA (Figs. 1b and c). The growth rate of presenescent TIG-1 cells with reduced *SMARCD1* expression was shown to be significantly decreased (Fig. 1d). Furthermore, we determined cellular senescence induction by analyzing cell size and the percentage of cellular senescence marker-positive cells, using a fully automated and robust cellular image-analysis system. Results show that the relative number of cells with increased senescence-associated β-galactosidase (SA-β-Gal) activity, p16/p21 expression, and phospho p38 (p-p38) expression is increased in *SMARCD1*-silenced TIG-1 cells (Fig. 1e).

In contrast, cellular senescence was shown to be suppressed in senescent TIG-1 cells (61 PDL) where *SMARCD1* was ectopically expressed (Fig. 1f), as evidenced by a decreased number of senescence marker-positive cells (Fig. 1h) in *SMARCD1*-overexpressing TIG-1 cells. Furthermore, *SMARCD1* expression attenuated the replicative senescence-induced growth retardation (Fig. 1g).

### Palmitic acid induces cellular senescence concurrently with the accumulation of intracellular lipids in hepatocytes

**Fig. 2:**
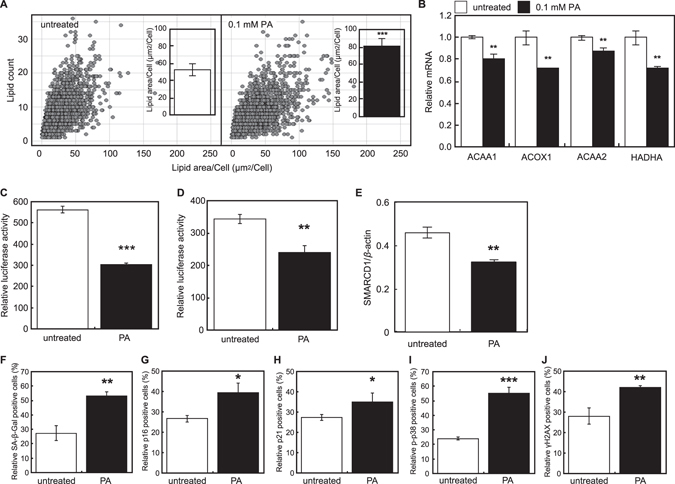
Palmitic acid induces cellular senescence and accumulation of lipid in hepatocytes. (**a**) HepG2 cells were cultured in the absence or presence of 0.1 mM palmitic acid, and the number of lipid droplets and the lipid area were quantified. Average lipid area was shown as a bar chart. (**b**) Relative expression of ACAA1, ACOX1, ACAA2, and HADHA in HepG2 cells cultured in the absence or presence of 0.1 mM palmitic acid. PPAR-dependent transcription (**c**) and transcriptional activation ability of PGC-1α (**d**) were assessed. (**e**) *SMARCD1* relative gene expression in HepG2 cells in the absence or presence of 0.1 mM palmitic acid (PA). Activity and expression of senescence markers (F, SA-β-Gal; G, p16; H, p21; I, phosphor p38; J, γH2AX) were measured in HepG2 cells cultured in the absence or presence of 0.1 mM PA. Data were analyzed by two-sided Student’s *t*-test and are expressed as mean ± SD. *P* < 0.05 was considered significant (^*^ *P* < 0.05; ^**^ *P* < 0.01; ^***^ *P* < 0.001)

As reported previously^8^, significant lipid droplet accumulation can be observed in HepG2 cells following the treatment with 0.1 mM palmitic acid (PA) (Fig. 2a). Furthermore, PA treatment led to a decrease in the expression of fatty acid oxidation (FAO) genes, including acetyl-CoA acyltransferase 1 (ACAA1), acyl-CoA oxidase 1, palmitoyl (ACOX1), ACAA2, and hydroxyacyl-CoA dehydrogenase (HADHA) (Fig. 2b). PA treatment induced the inactivation of PGC-1α (Fig. 2c) and repressed the transcriptional activation ability of PGC-1 (Fig. 2d), determined by the repressed PPRE-dependent transcription and PGC-1α-GAL4-dependent transcription, respectively.

Additionally, PA treatment led to a reduction of *SMARCD1* expression in HepG2 cell (Fig. 2e), and induced cellular senescence in HepG2 cells, demonstrated by an increased number of cells with high levels of SA-β-Gal activity, p16/p21 expression, p-p38 expression, and γH2AX expression in the PA-treated HepG2 cells. Senescent cells express reduced level of SMARCD1 as shown in Fig. 1a, suggesting that senescence-inducing signals reduce the SMARCD1 expression. Palmitic acid reduces the SMARCD1 expression possibly because of its cellular senescence inducing ability.

### The role of SMARCD1 in lipid accumulation and senescence induction in HepG2 cells

**Fig. 3:**
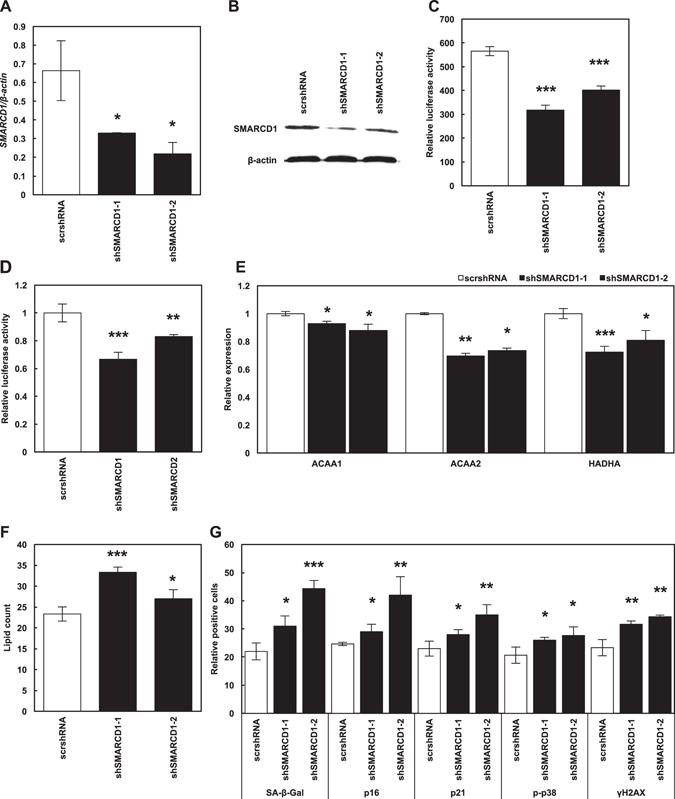
Lipid accumulation and senescence induction in *SMARCD1*-silenced HepG2 cells. (**a**) *SMARCD1* relative expression in SMARCD1-silenced HepG2 cells. (**b**) Expression of SMARCD1 in scr-shRNA-transduced and SMARCD1-silenced HepG2 cells. PPAR-dependent transcription (**c**) and transcriptional activation ability of PGC-1α (**d**) in scr-shRNA-transduced and SMARCD1-silenced HepG2 cells. (**e**) Relative expression of FAO genes in scr-shRNA-transduced and *SMARCD1*-silenced HepG2 cells. (**f**) The number of lipid droplet in scr-shRNA-transduced and *SMARCD1*-silenced HepG2 cells. (**g**) Activity and expression of senescence markers were measured in scr-shRNA-transduced and SMARCD1-silenced HepG2 cells. Data are expressed as mean ± SD. *P* < 0.05 was considered statistically significant (^*^ *P* < 0.05; ^**^ *P* < 0.01; ^***^ *P* < 0.001)

We specifically targeted *SMARCD1* expression in HepG2 cells using shRNA (Figs. 3a and b). In *SMARCD1*-silenced HepG2 cells, PGC-1α activity and its transcriptional activation ability were both shown to be attenuated (Figs. 3c and d). Recruitment of PGC-1α-SMARCD1 complex to PPARα-binding site would activate the promoter having PPAR response element such as PGC-1α promoter, which suggest that *SMARCD1* knockdown downregulated the transcriptional activity of PGC-1α^9^. Furthermore, in *SMARCD1*-silenced HepG2 cells, FAO gene expression levels were downregulated (Fig. 3e), indicating a significant accumulation of lipid droplets (Fig. 3f). In contrast, the expression of cellular senescence markers were shown to be increased in these cells (Fig. 3g).

**Fig. 4:**
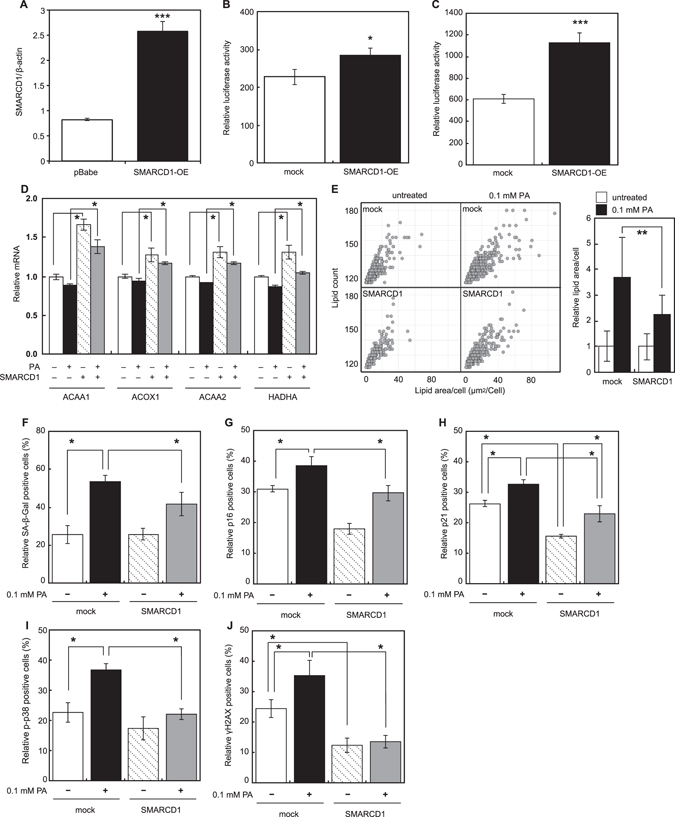
Lipid accumulation and senescence induction in the *SMARCD1*-overexpressing HepG2 cells cultured in the absence or presence of 0.1 mM PA. (**a**) Relative *SMARCD1* expression in SMARCD1-overexpressing HepG2 cells. PPAR-dependent transcription (**b**) and transcriptional activation ability of PGC-1α (**c**) were determined in SMARCD1-overexpressing HepG2 cells. (**d**) Relative expression of FAO genes in mock-transduced and *SMARCD1*-overexpressing HepG2 cells in the absence or presence of PA. (**e**) The number of lipid droplets and lipid area in mock-transduced and *SMARCD1*-overexpressing HepG2 cells in the absence or presence of PA. Activity and expression of senescence markers (F, SA-β-Gal; G, p16; H, p21; I, phosphorylated p38; J, γH2AX) were measured in mock-transduced and *SMARCD1*-overexpressing HepG2 cells. Data are expressed as mean ± SD (^*^ *P* < 0.05; ^**^ *P* < 0.01; ^***^ *P* < 0.001). Multiple comparisons between groups were made by one-way ANOVA with Tukey’s post hoc test. Statistical significance was defined as *P* < 0.05 (^*^ *P* < 0.05; ^**^ *P* < 0.01)

Furthermore, we overexpressed *SMARCD1* in HepG2 cells (Fig. 4a), and noticed a significantly increased PGC-1α activity and transcriptional activation ability (Figs. 4b and c). In the absence or presence of PA treatment, *SMARCD1* overexpression led to a significant increase in FAO gene expression (Fig. 4d). Additionally, PA-induced accumulation of lipid droplet decreased in cell overexpressing *SMARCD1* (Fig. 4e).

Next, we evaluated the role of *SMARCD1* in the PA-induced cellular senescence in HepG2 cells. PA-induced cellular senescence was shown to be attenuated in *SMARCD1*-overexpressing HepG2 cells, demonstrated by the inhibition of SA-β-Gal activity, p16/p21 expression, p-p38 expression, and γH2AX expression (Figs. 4f–j).

### Involvement of Smarcd1 in high-fat diet (HFD) induced hepatic lipid accumulation

**Fig. 5:**
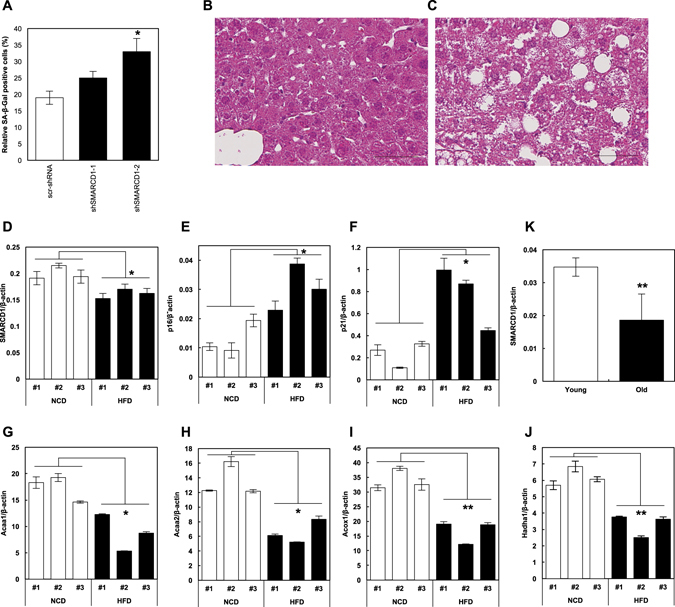
Smarcd1 roles in high-fat diet-induced hepatic lipid accumulation. (**a**) Senescence induction in scr-shRNA-transduced and Smarcd1-silenced normal mouse primary hepatocytes. Hematoxylin-eosin staining of liver sections obtained from 20-week-old mice fed with normal chow diet (NCD) (**b**) or high-fat diet (HFD) (**c**). Scale bar, 50 μm. Relative gene expression of *Smarcd1* (**d**), p16 (**e**), p21 (**f**), and FAO genes (**g**–**j**) in livers obtained from NCD or HFD mice. Number indicates the individual mice. (**k**) Relative expression of SMARCD1 gene in peripheral blood mononuclear cells derived from young and old volunteers. Data are expressed as mean ± SD. *P* < 0.05 was considered statistically significant (^*^ *P* < 0.05; ^**^ *P* < 0.01)

Firstly, we evaluated the effect of *Smarcd1*-knockdown on the cellular senescence induction in mouse primary hepatocytes, indicating that *Smarcd1* knockdown also induced cellular senescence in normal hepatocytes (Fig. 5a). Experimental animals were fed a HFD, and the subsequent hematoxylin-eosin staining of their liver sections revealed the accumulation of lipids, in contrast with mice that received normal chow diet (NCD) (Figs. 5b and c)^10^. Using this experimental model of hepatic lipid accumulation, we elucidated Smarcd1 roles in the cellular senescence and lipid accumulation. The obtained results demonstrated that FAO gene expression levels were significantly downregulated in mice that received HFD (Figs. 5g–j). Furthermore, *Smarcd1* expression was downregulated (Fig. 5d) and the expression of senescence markers (p16 and p21) was upregulated in these mice (Figs. 5e and f).

Finally, we tracked the changes in expression level of *SMARCD1* during the aging process by using mRNA of peripheral blood mononuclear cells (PBMC) from young and old volunteers. Result showed that *SMARCD1* expression decreased in PMBC of old volunteers (Fig. 5k), suggesting that decreased expression of *SMARCD1* is one of reasons for the occurrence of aging-associated diseases.

## Discussion

Previously, we identified SMARCD1 as a senescence-associated gene by a subtractive proteomic analysis^4^, and here we elucidated the roles of SMARCD1 as a senescence-associated molecule. Because the deregulation of SWI/SNF function is reported to promote cellular senescence, dysfunction of SMARCD1, a member of SWI/SNF family protein, is thought to induce cellular senescence through regulation of chromatin remodeling and gene transcription^11, 12^. SMARCD1 is involved in physiological regulation of hepatic fat oxidation^9^ and plasma cholesterol levels^13^. Here, we focused on PGC-1α-mediated SMARCD1 roles in the fatty acid oxidation and PA-induced hepatic senescence.

We demonstrated that the inhibition of *SMARCD1* expression induces cellular senescence in presenescent TIG-1 cells, and induces the accumulation of lipid droplets through the inactivation of PGC-1α-dependent transcription and suppressing FAO gene expression, consistent with the previous reports, showing that adenovirus-mediated expression of *SMARCD1* stimulates FAO gene expression in hepatocytes and ameliorates hepatic steatosis *in vivo* ^9^. These results suggest that *SMARCD1*-inhibiting signals can trigger the accumulation of lipid droplets and induction of steatosis, showing that SMARCD1 plays a pivotal role in the suppression of lipid droplet accumulation and cellular senescence induction.

Li et al. and we showed that saturated fatty acids, such as PA, and HFD reduce the expression of *SMARCD1* possibly through the epigenetic modifications, because PA treatment of human pancreatic islets gives rise to epigenetic modifications of several genes, which may contribute to altered gene expression^14^. This reduced expression of *SMARCD1* consequently induce the accumulation of lipid droplets *in vitro* and the development of fatty liver *in vivo* ^9^. The results obtained in this study show that PA also induces cellular senescence in HepG2 cells, further indicating a relationship between lipid droplet accumulation and cellular senescence induction in HepG2 cells. We showed that *SMARCD1* functions as a suppressor of PA-induced cellular senescence in HepG2 cells.

Taken together, our results firstly indicate that SMARCD1 functions as an inhibitor of hepatic senescence. Several reports have investigated the roles of senescent hepatic stellate cells, and showed that senescence may be induced in response to liver damage, limiting fibrogenic response to acute tissue damage by degrading extracellular matrix and enhancing immune surveillance^15^. Furthermore, deoxycholic acid (DCA) produced by gut microbiota of obese mice was reported to induce cellular senescence and to provoke senescence-associated secretory phenotype (SASP) in hepatic stellate cells via enterohepatic circulation, which in turn secrete various inflammatory cytokines and tumor-promoting factors in liver and facilitate hepatocellular carcinoma development in mice^16^. Therefore, senescent hepatic stellate cells show distinctive phenotypes that depend on cellular milieu and senescence-inducing stimuli. Hepatocyte senescence has been demonstrated in chronic liver diseases, including insulin resistance and fibrosis scarring at the cirrhosis^17, 18^. Furthermore, it was reported to correlate closely with fibrosis stage and diabetes mellitus, as reported in the study using liver sections from patients with non-alcohol-related fatty liver disease (NAFLD)^19^. In this study, we firstly report that hepatocyte senescence is closely related to the lipid accumulation, and that SMARCD1 is a key molecule in both processes. Inactivation of PGC-1α by decreased SMARCD1 expression would explain the simultaneous induction of cellular senescence and lipid accumulation^20^. Signals that augment the expression of SMARCD1 prevent lipid accumulation through the activation of β-oxidation and inhibition of cellular senescence, and suppress the functional decline of liver.

Intriguing reports regarding the role of SMARCD1 in lipid and cholesterol metabolisms demonstrated that a decrease in the expression of SMARCD1 induced by HFD suppresses fatty acid β-oxidation and induces hepatic steatosis^9, 21^, while the so-called “Western diet” (high fat and high cholesterol contents) induces SMARCD1 expression, which results in the stimulation of the expression of genes involved in bile acid synthesis, modification, and transport, elevation of plasma cholesterol level, and hyperlipidemia^13^. In other words, fasting and high fat/cholesterol diet both induce SMARCD1, but suppress steatosis and induce hypercholesterolemia/atherosclerosis, respectively. Phenotypic differences induced by SMARCD1 would be determined by cofactors binding to SMARCD1, PGC-1α and constitutive androstane receptor (CAR). Regulation mechanisms of PGC-1α and CAR depending upon diet condition must be clarified in the future study.

These results indicate that dietary nutrients interact with gene networks, orchestrating the adaptive response, where SMARCD1 plays a pivotal role, suggesting that the control of diet-sensitive SMARCD1 levels is necessary for the prevention of lifestyle-related diseases.

## Methods

### Cell culture and reagents

TIG-1 cells (human normal diploid fibroblast, Cell Resource Center for Biomedical Research, Institute for Development, Aging and Cancer, Tohoku University, Miyagi, Japan) and HepG2 cells (human cell line derived from hepatocyte carcinoma, RIKEN BRC, Tsukuba, Japan) were cultured in MEM medium (Nissui, Tokyo, Japan) and DMEM medium (Nissui), respectively, supplemented with 10% fetal bovine serum (FBS, Life Technologies, Gaithersburg, MD, USA) at 37 °C in a 95% air/5% CO_2_ atmosphere. PA was purchased from Chem Service (West Chester, PA, USA). TIG-1 cells (37 PDL) and (61 PDL) were used as presenescent and senescent cells, respectively.

### Fatty acid treatment

PA complexed with bovine serum albumin (BSA) was prepared as described previously^8^.

### Quantification of intracellular lipid droplets

HepG2 cells were fixed with 10% formaldehyde for 10 min at room temperature. After washing the cells with PBS, they were stained with 0.06 μg/mL BODIPY 493/503 (Life Technologies) and 1 μg/mL Hoechst 33342 solution (Dojindo, Kumamoto, Japan), in order to stain intracellular lipid and nucleus, respectively. Fluorescence images were obtained using IN Cell Analyzer 1000 (GE Healthcare, Amersham Place, UK), analyzed using IN Cell Developer Toolbox 1.9 (GE Healthcare), and visualized by Spotfire DecisionSite Client 8.2 software (PerkinElmer, Waltham, MA, USA).

### Fluorescence SA-β-gal assay

Fluorescence SA-β-Gal assay was conducted according to a method reported previously^22^. Briefly, cells seeded onto μClear Fluorescence Black Plate (Greiner Bio-One, Tokyo, Japan) were fixed with 2% formaldehyde/0.2% glutaraldehyde, and stained with PBS (pH 6.0) containing 33 μM ImaGene Green C_12_FDG (Life Technologies) and 1 μg/mL Hoecst 33342. The images were acquired using IN Cell Analyzer 1000, and analyzed using IN Cell Developer Toolbox 1.9. The imaging data were reported as SA-β-Gal intensity (mean fluorescence intensity per cell) and the number of SA-β-Gal positive/negative cells. The threshold of SA-β-Gal intensity was set so that about 75% of all control cells were negative.

### Immunofluorescence

Cells were fixed with 4% paraformaldehyde for 15 min at room temperature, and permeabilized with ice-cold methanol at −20 °C for 10 min. After washing the cells, cells were blocked with blocking solution (5% goat serum, 0.3% Triton-X 100) for 1 h. After removing the solution, cells were incubated with primary antibodies (anti-p16^INK4a^ (ab81278; Abcam, Cambridge, MA, USA); ant-p21 (#2947; Cell Signaling, Beverly, MA, USA); anti-phospho-p38 MAPK (#4511; Cell Signaling); anti-phospho-histone H2A.X (#9718; Cell Signaling)) at 4 °C overnight. After washing the cells, they were next incubated with the secondary antibody (Alexa Fluor 555 F(ab’) fragment of goat anti-rabbit IgG; Life Technologies) at room temperature for 1 h. Following another washing, cells were incubated with 1 μg/mL of Hoechst 33342 solution at room temperature for 10 min. The images were analyzed using the IN Cell Developer Toolbox 1.9, and the results were depicted using SpotFire DecisionSite Client 8.2 Software.

### Quantitative PCR (qPCR)

RNA was prepared using the High Pure RNA Isolation Kit (Roche Diagnostics GMBH, Mannheim, Germany), and cDNA was prepared using ReverTra Ace (Toyobo, Osaka, Japan), according to the manufacturer’s instructions^23, 24^. qPCR was performed using the KAPA SYBR Fast qPCR Kit (KAPA Biosystems, Woburn, MA, USA) and Thermal Cycler Dice Real Time System TP-800 (TaKaRa, Shiga, Japan). The samples were analyzed in triplicate, and *SMARCD1* levels were normalized using β-actin levels. PCR primer sequences were described elsewhere (Supplementary Table 1).

### Immunoblotting

Cell lysates were resolved by electrophoresis using 12% SDS-PAGE and transferred to a Hybond P membrane (GE Healthcare). The membrane was incubated with anti-SMARCD1 antibody (10998-2-AP; Proteintech, Rosemont, IL) or anti-β-actin (013-24553; Applied Biological Materials Inc., Richmond, BC, Canada). Horseradish peroxidase-labeled anti-rabbit IgG antibody (GE Healthcare) and ant-mouse IgG antibody (GE Healthcare) was used as secondary antibodies. The proteins were detected using ImmunoStar LD (Wako Pure Chemical, Osaka, Japan) and visualized with a LAS-1000 Lumino Image analyzer (Fuji Film, Tokyo, Japan).

### Retrovirus production and transduction

Retrovirus production and transduction was described previously^23^. Target cells were infected with this viral supernatant for 24 h at 37 °C, and then were selected using 3 μg/mL puromycin (Enzo Fife Sciences, Farmingdale, NY) for 3 days.

### Short hairpin RNA (shRNA)

Oligonucleotides containing siRNA-expressing sequence targeting *SMARCD1* were annealed (shSMARCD1 top: 5’-GATCCCCGCAGATCTTTGAGTCTCAACGTTCGAAGAGCGTTGAGACTCAAAGATCTGCTTTTTA-3’, shSMRCD1 bottom: 5’-AGCTTAAAAAGCAGATCTTTGAGTCTCAACGCTCTTCGAACGTTGAGACTCAAAGATCTGCGGG-3’; shSMARCD2 top: 5’-GATCCCCGCAGGGACCTCAAGACAATGATTCGAAGAGTCATTGTCTTGAGGTCCCTGCTTTTTA-3’, shSMARCD1 bottom: 5’-AGCTTAAAAAGCAGGGACCTCAAGACAATGACTCTTCGAATCATTGTCTTGAGGTCCCTGCGGG-3’), and cloned into the pSUPER.retro vector (OligoEngine, Seatle, WA, USA).

### Promoter assay

PPRE × 3-TK-luc containing 3 × DR1 sites was a kind gift provided by Bruce Spiegelman (Addgene plasmid #1015) and was used as reporter^25^. HepG2 cells (24-well plates) were transfected with reporter constructs using HilyMax reagent according to the manufacturer’s protocol^23^. Luciferase assay was performed using the Dual-Luciferase Reporter Assay System (Promega, Madison, WI, USA). The relative luciferase activity was calculated by dividing the firefly luciferase activity with the Renilla luciferase activity.

To evaluate the transcriptional activation ability of PGC1α, we used GAL4-PGC1α, containing a full-length PGC1α fused to the GAL4 DNA binding domain, kindly provided by Bruce Spiegelman (Addgene plasmid #8892)^26^. The luciferase assay was performed using GAL4-PGC1α and pFR-Luc (Agilent Technologies, Santa Clara, CA, USA).

### Animal experiments

Animal experiments were performed as described previously^10^.C57/BL6J mice (male, *n* = 3) were fed *ad libitum* with a NCD (5.4% fat, CRF-1; Orient Yeast, Tokyo, Japan). Additionally, mice (male, *n* = 3) were provided with an HFD (24% fat, lard fat, 188.28 kj% fat, D12451; Research Diets, New Brunswick, NJ, USA) for 16–24 weeks. In this experiment, randomization and blinding were not adopted. For histochemical analyses, the isolated livers were fixed, processed, and stained with hematoxylin-eosin. All mouse experiments and protocols were in accordance with the Guide for the Care and Use of Laboratory Animals and were approved by the Ethics Committees on Animal Experimentation (Kyushu University, Graduate School of Medicine, Japan).

### RNA extraction from whole blood samples

Volunteers were grouped into two categories based on age (Young group: 24.0 ± 0.8 years old (*n* = 4); Old group: 69.0 ± 2.8 years old (*n* = 4)). Whole blood was collected by PAXgene Blood RNA collection tubes (Qiagen, Valencia, CA). RNA was extracted from peripheral blood mononuclear cells by using a PAXgene RNA kit according to the manufacturer’s instructions (Qiagen). cDNA synthesis and qRT-PCR were performed as described as above. The study was approved by the Ethics Committees of Kyushu University. Written informed consent was obtained from all participants.

### Statistical analysis

All experiments were performed at least 3 times, and the corresponding data are shown. All data are expressed as mean ± standard deviation (SD) and normally distributed variables were evaluated by two-sided Student’s t test (^*^ *P* < 0.05; ^**^ *P* < 0.01; ^***^ *P* < 0.001). Multiple comparisons between groups were made by one-way ANOVA with Tukey’s post hoc test. Statistical significance was defined as *P* < 0.05 (^*^ *P* < 0.05; ^**^ *P* < 0.01).

### Data availability statement

The data sets generated during and/or analysed during the current study are available from the corresponding author on reasonable request.

## Electronic supplementary material

Supplementary Table 1

## Electronic supplementary material

**Supplementary Information** accompanies the paper on the *npj Aging and Mechanisms of Disease* website (doi:10.1038/s41514-017-0011-1).
