# Supplementary material for: SMARCD1 regulates senescence-associated lipid accumulation in hepatocytes
Source: NPJ Aging Mech Dis. 2017 Aug 30;3:11. doi: 10.1038/s41514-017-0011-1 (PMC5577293; doi:10.1038/s41514-017-0011-1)
Supplement: Supplementary file 1 — Supplementary Table 1 [file 41514_2017_11_MOESM1_ESM.docx]

**Supplementary Table 1 Primers**

| Species | Name | Sequence |
| --- | --- | --- |
| Homo sapiens | β-actin | 5’-TGGCACCCAGCACAATGAA-3’ |
|  |  | 5’-CTAAGTCATAGTCCGCCTAGAAGCA-3’ |
| Homo sapiens | SMARCD1 | 5’-AGACCAGGTATGTTGCCAGG-3’ |
|  |  | 5’-ACTGATCCATCCCTGACTGG-3’ |
| Homo sapiens | ACAA1 | 5’-CTGAGAAGGTGAACCCCCTG-3’ |
|  |  | 5’-CACTCCGTATGCCCTCTTCC-3’ |
| Homo sapiens | ACOX1 | 5’-AAGGCGACATCAATCCGAAC-3’ |
|  |  | 5’-CTACGAATGGGAAGGAATGGAA-3’ |
| Homo sapiens | ACAA2 | 5’-GTCTGCTGGCAAAGTCTCACC-3’ |
|  |  | 5’-ATTCCCACACGCAAACCAA-3’ |
| Homo sapiens | HADHA | 5’-AGGAAGGAGTTGACCCGAAGA-3’ |
|  |  | 3’-CAAACCGCTCCCCAAAGA-3’ |
| Homo sapiens | PGC-1α | 5’-GCTGACAGATGGAGACGTGA-3’ |
|  |  | 5’-TAGCTGAGTGTTGGCTGGTG-3’ |
| Homo sapiens | p16 | 5’-GGCACCAGAGGCAGTAACCA-3’ |
|  |  | 5’-GGACCTTCGGTGACTGATGATCTAA-3’ |
| Homo sapiens | p21 | 5’-TCAAATCGTCCAGCGACCTTC-3’ |
|  |  | 5’-GTCCATAGCCTCTACTGCCACCA-3’ |
| Mouse | β-actin | 5’-GGCCAGGTCATCACTATTG–-3’ |
|  |  | 5’-GAGGTCTTTACGGATGTCAAC– -3’ |
| Mouse | Smarcd1 | 5’-TGGTGATCGAACTGGACAAA-3’ |
|  |  | 5’-CCAGCATCAGCAGGACAGTA-3’ |
| Mouse | Acaa1 | 5’-TGAGCGGTTTGGCGTTT-3’ |
|  |  | 5’-CTTGTCACCCTTGTCATTCAGG-3’ |
| Mouse | Acox1 | 5’-TATGGGATCAGCCAGAAAGGA-3’ |
|  |  | 5’-AAAGTCAAAGGCATCCACCAA-3’ |
| Mouse | Acaa2 | 5’-TGCCCCTCAGTTCTTGTCTGT– -3’ |
|  |  | 5’-AGGTGTGCGGTGATTCTGG-3’ |
| Mouse | Hadha | 5’-GTGATTGAGGCTGTGTTTGAGG-3’ |
|  |  | 5’-TTTGATTGATTGGGAGAGCAGA-3’ |
| Mouse | p16 | 5’-CATCGTGCGATATTTGCGTT-3’ |
|  |  | 5’-GAGCTGAAGCTATGCCCGTC-3’ |
| Mouse | p21 | 5’-GCAAAGTGTGCCGTTGTCTC-3’ |
|  |  | 5’-CGTCTCCGTGACGAAGTCAA-3’ |
